# Bubble cascade in Guinness beer is caused by gravity current instability

**DOI:** 10.1038/s41598-019-42094-0

**Published:** 2019-04-05

**Authors:** Tomoaki Watamura, Fumiya Iwatsubo, Kazuyasu Sugiyama, Kenichiro Yamamoto, Yuko Yotsumoto, Takashi Shiono

**Affiliations:** 1 Graduate School of Engineering Science, Osaka University, 1-3, Machikaneyama, Toyonaka, Osaka 560-8531 Japan; 2 Research Laboratory for Beverage Technologies, Research & Development Division, Kirin Co. Ltd., 1-17-1, Namamugi, Tsurumi-ku, Yokohama, Kanagawa 230-6826 Japan

## Abstract

The downward movement of the bubble-texture in a glass of Guinness beer is a fascinating fluid flow driven by the buoyant force of a large number of small-diameter bubbles. This texture motion is a frequently observed phenomenon on pub tables. The physical mechanism of the texture-formation has been discussed previously, but inconsistencies exist between these studies. We performed experiments on the bubble distribution in Guinness poured in an inclined container, and observed how the texture forms. We also report the texture-formation in controllable experiments using particle suspensions with precisely specified diameters and volume-concentrations. Our specific measurement methods based on laser-induced-fluorescence provide details of the spatio-temporal profile of the liquid phase velocity. The hydrodynamic condition for the texture-formation is analogous to the critical point of the roll-wave instability in a fluid film, which can be commonly observed in water films sliding downhill on a rainy day. Here, we identify the critical condition for the texture-formation and conclude that the roll-wave instability of the gravity current is responsible for the texture-formation in a glass of Guinness beer.

## Introduction

Following Archimedes’ principle, bubbles in liquid generally rise because of the gas-liquid density difference. Despite the natural rising behaviour of bubbles, after pouring Guinness beer in a pint glass, the bubbles can be observed to descend. At the same moment, a vast number of small bubbles with a mean diameter of 50 μm (only 1/10 the size of those in Budweiser or champagne^1–3^) disperse throughout the entire glass. We can also observe the fascinating texture motion as a number-density distribution of bubbles travelling downwards. Curiously, although creamy bubbles have been served in Guinness beer for more than half a century, the mystery of such a cascading motion of bubbles has been debated in terms of fluid dynamics ever since^4–7^. Because the black colour of Guinness obstructs the physical observation in a glass, computational simulations have been a valuable tool to understand the bubble distribution and motion. The computational investigation has concluded that when Guinness is poured into a typical pint glass, which widens towards its top, the rising motion of bubbles creates a clear-fluid (bubble-free) film above the inclined wall. The dense clear-fluid film falls, whereas the bubble-rich bulk rises^7^, which is known as the Boycott effect^8^. Accordingly, we can observe the descending bubbles entrained into the downward flow in Guinness, which is seemingly paradoxical in light of Archimedes’ principle. Although this physical explanation leads to the understanding of the descending motion of bubbles, the mechanism underlying the texture-formation still remains an open problem.

The texture motion of bubble swarm moving downwards, so-called “*waves*” or “*cascades*”, is a unique and frequently-observed phenomenon in a glass of stout beer with nitrogen. Analogies to the texture-formation in Guinness have been noted in several studies^4,5^. The reduction in the rise velocity of bubbles in large bubble-volume-concentration fluids, known as the hindered velocity^9,10^, was suggested to be one of the mechanisms, i.e., the velocity difference in the bubble swarm is involved in the downward movement of the bubble swarm as waves, even when the buoyant bubbles themselves rise^4^. On the other hand, a one-dimensional flow model, which assumes an eddy viscosity due to the variation in bubble-volume-concentration, was developed to include a mathematical structure of the roll-wave instability, which can be commonly observed in water films sliding downhill on rainy days^5^. However, in previous studies, there have been inconsistencies in the movement directions and in the longitudinal wavelengths^4,5,7^. Moreover, the presence of a clear-fluid film was not considered in the model^5^, and the hydrodynamic condition for the texture-formation have not been discussed so far. In this study, we propose a falling liquid film model assuming stratified two-fluids, and we experimentally quantify the critical condition for the roll-wave formation involved with the hydrodynamic instability in the gravity current in the inclined wall vicinity.

## Experiments

### Experimental apparatus

**Figure 1:**
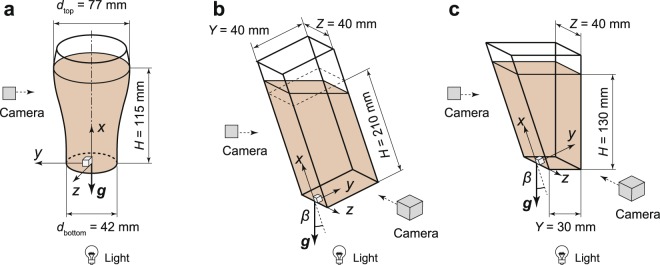
Schematic of the experimental configuration. (**a**) A pint glass; (**b**) inclined rectangular container; and (**c**) trapezium container.

We studied the motion of bubbles in three different containers: a pint glass (Fig. 1a), an inclined rectangular container (Fig. 1b), and a trapezium container (Fig. 1c). The inclination angle *β* was adjusted from 0 degrees (vertical) to 70 degrees. To observe the motion of the bubble-texture, we poured Guinness beer in the containers from a 330-ml can of Draught Guinness. Note that we measured the density and viscosity of liquid in Guinness beer ourselves and found that the liquid density was *ρ*_0_ = 1006 kg/m^3^ and viscosity was *μ* = 2.1 × 10^−3^ Pa·s (see also Supplementary Fig. S1). The mean diameter of bubbles in Guinness was 61 μm, and the volume-concentration of the bubbles was *α* = 8%. The details of the fluid properties are also summarised in Supplementry Table 1.

### Pseudo-Guinness fluid

To conduct well-controlled experiments, we also used a particle suspension comprising tap water with a mean temperature of 25 °C (*μ* = 0.9 × 10^−3^ Pa·s) and hollow spherical glass particles with a mean diameter of 47 μm and a density of *ρ*_1_ = 140 kg/m^3^. The volume-concentration of glass particles *α* was adjusted to 0.5–10%, making it comparable with the volume-concentration of bubbles in Guinness. By using undeformable glass particles, we could neglect the expansion of the bubble diameter due to the diffusion of mixture gas, comprising CO_2_ and N_2_, from the supersaturated solution into the bubble interior^4,6^. We could also neglect the modification of the rise motion due to a nonuniform contamination distribution of beer components along the bubble surface, known as the Marangoni effect^11^. The particle suspension offers another striking advantage: it is suitable for measuring the liquid phase velocity in the transparent liquid phase of suspension rather than the opaque Guinness. Consequently, for measuring the liquid phase velocity, we could employ optical measurement instruments applying laser-induced-fluorescence.

### Flow visualisation using molecular tags

**Figure 2:**
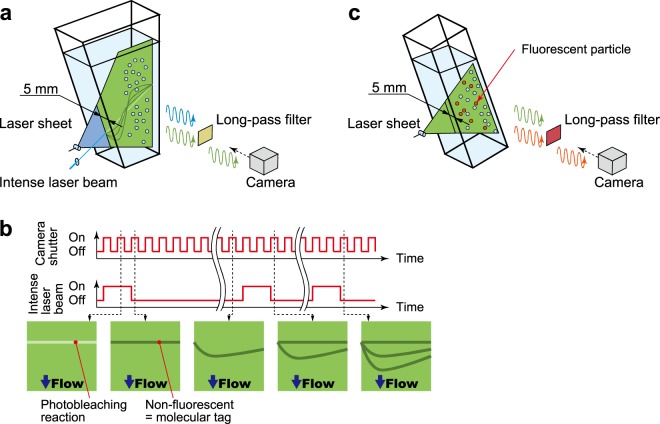
(**a**) Schematic of the visualisation setup and (**b**) diagram of the tag formation for the photo-bleaching molecular tagging method. The irradiation interval and duration of the intense laser were controlled by a function generator. (**c**) Schematic of the setup for velocity measurement via PIV applying LIF. In the both systems, a long-pass optical-filter was installed on a high-speed video-camera, to reduce reflectance from particles and detect fluorescence emitted by LIF-particles.

The photo-bleaching molecular tagging visualisation^12,13^, which is the elaborated non-contact and non-invasive measurement technique for the liquid-phase of bubbly flows, was applied for the quantitative visualisation of the liquid phase velocity. Figure 2a shows a schematic of the visualisation setup. Fluorescein was dissolved in tap water with *O*(10^−6^) mol/m^3^. An intense laser beam at a wavelength of 447 nm was focused with 250 μm beam waist diameter using a convex lens, and was irradiated on the measurement (*x*-*y*) plane of the trapezium container. A 1 mm thick laser sheet at wavelength of 447 nm which is installed 5 mm away from the vertical wall illuminated the measurement plane. The irradiation time and the time interval between two-consecutive irradiations of intense laser beams were adjusted to 20 ms and 100 ms for *α* = 0.5%, whereas 100 ms and 200 ms for *α* = 5%, respectively, using a function generator. As illustrated in Fig. 2b, the molecular tag formed by the intense laser beam appears as a dark region, i.e., non-fluorescent region, on the measurement plane. The displacement and distortion of the molecular tags inform us of the liquid phase flow pattern as a timeline image. The reflected light at 447 nm from the particle interface was blocked by an optical long-pass filter with a wavelength range of 480 ± 5 nm, whereas most of the fluorescent light passed the filter. Therefore, the tag lines being the non-fluorescent region were clearly visualised in fluorescent images.

### Measurement of the liquid phase velocity

The velocity of the liquid phase was obtained via direct cross-correlation particle image velocimetry (PIV)^14^. To distinguish tracer particles in particles suspension, we applied the laser-induced-fluorescence (LIF) technique to PIV^15^. Fluorescent tracer particles with 15 μm in diameter (specific gravity: 1.1, absorption wavelength: 550 nm, emission wavelength: 580 nm) were seeded at 0.03 vol. % in the particle suspension. A diode-pumped-solid-state laser at wavelength of 532 nm illuminated a light sheer at *x* ≈ 100 mm with 1 mm thickness in the rectangular container. The reflected light at 532 nm from the particle interface was blocked by an optical long-pass filter with a wavelength range of 580 ± 5 nm, whereas most of the fluorescent light passed the filter (Fig. 2c). We treated bubbles in Guinness as behaving like tracer particles. The outlier velocity vectors were eliminated by comparing with the surrounding vectors^16^, and any missing vectors were interpolated.

## Results

### Inclined angle dependency on texture formation

**Figure 3:**
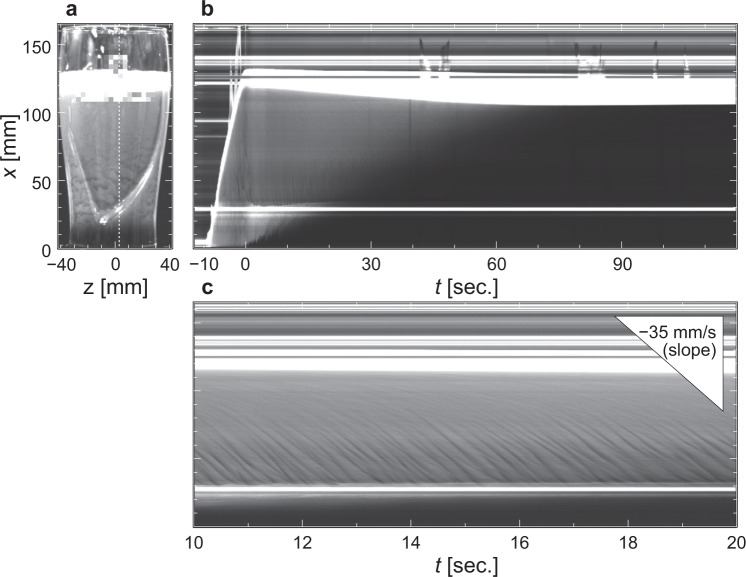
(**a**) A snapshot of the bubble-concentration texture of Guinness beer in the pint glass. The corresponding movie is presented in the Supplementary Movie S1. (**b**), Temporally expanded image of the bubble-concentration distribution at the white dotted vertical line in Fig. 3a (near the centreline of the pint glass). (**c**) Enlargement of (**b**) showing travelling waves in the downward direction. The slope of the triangle shown at the upper right corresponds to reference value of −35 mm/s for the maximum downward velocity of the wave^5^.

We start by showing qualitative features of the texture-formation in a pint glass. Figure 3a shows a snapshot of the texture as the number-density distribution of bubbles in a pint glass 10 seconds after pouring Guinness. Visually, the texture can be easily observed at 30 mm < *x* < 70 mm, where the wall is inclined due to the tapered shape of the glass with inclined angle of ≈ 15 degrees. Figure 3b shows the temporal change in the phase separation: the black region (liquid of beer), the grey region (bubbly flow), and the white region (head of foam). The bubbly flow region gradually decreases with increasing time and the amount of foam head increases. Finally, the glass of Guinness is completely separated into two contrasting phases (namely, the liquid and the head of foam), corresponding to the phase separation owing to the buoyant rise of each bubble. The texture appearing in a glass propagates downwards with a travelling velocity of ~−35 mm/s (Fig. 3c, Supplementary Movie S1), which is consistent with previous experimental observation^5^. Because of the differences in the travelling velocity of each wave and the three dimensionality of the flow, the neighbouring waves collide and coalesce, and thus the diagonal stripes in the travelling-wave exhibit the bifurcations and crossovers in the texture.

**Figure 4:**
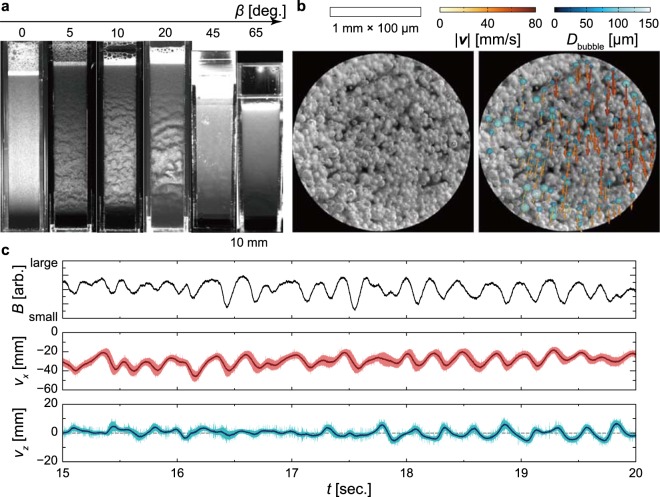
Texture-formation and motion of bubbles. (**a**) Snapshots of the bubble-concentration wave in the rectangular container for various inclination angles *β*. (**b**) Microscope image of bubbles in the trapezium container (left), and a superimposed display of bubbles extracted from the image by the template matching method^28^, showing the bubble-velocity vector obtained via the particle tracking method^29^ (right). Time-sequential images were captured by a high-speed video-camera mounted on a microscope. The corresponding movie is included as Supplementary Movie S2. (**c**) Temporal variation in the brightness of image *B* (top), the vertical *v*_*x*_ (middle) and horizontal *v*_*z*_ (bottom) bubble velocities. The shaded regions correspond to envelopes given by the local standard deviation of the bubble velocity in the measurement area.

To test the effect of inclination angle on the texture-formation, we repeated the observation of the texture-formation but after adjusting the inclination angle *β* of the rectangular container. The texture-formation result is shown in Fig. 4a. We found that the bubbles were uniformly distributed at *β* = 0, 45 and 65 degrees. For 5 degrees < *β* < 20 degrees, however, the texture appeared and exhibited spatial development: from organised waves to unstructured motions, similar to that of the Tollmien-Schlichting wave or the roll-wave^17,18^. This finding suggests that the appearance of the texture is triggered by the inclination of the wall and depends on the length of the system. In spatially developing flows, disturbances grow with distance from a flow entrance and can be clearly observed as wave at a sufficient distance to downstream. Although the texture was not clearly found at *β* > 45 degrees in Fig. 4a, it might possibly appear if the container were taller. We restrict ourselves to the study of the texture-formation in a glass with a height of ~150 mm (the typical size of pint glasses) and focus on the effect of inclination angle. To address the moving direction of bubbles yielding inconsistent results between previous investigations, i.e., whether bubbles move upwards^4^ or downwards^7^, we examined the velocity of bubbles near the inclined wall at *y* ≈ 0.5 mm and *x* ≈ 80 mm. The corresponding video can be viewed in Supplementary Movie S2. From the superimposed movie of bubble-diameter/velocity, we conclude that this approach provides an excellent measure for both the diameter and the velocity of bubbles. The instantaneous velocities are shown in Fig. 4b, and the time series of the brightness in image *B* involved with the local-volume-concentration of bubbles and the time series of bubble velocities are shown in Fig. 4c, respectively. The bubbles move downwards with both the vertical and horizontal fluctuations. The cross-correlation between the brightness *B* and the vertical component of the bubble velocity *v*_*x*_ is ≈0.8, exhibiting sufficiently high value. Such a high correlation indicates that bubbles are likely to move downwards more quickly in lower local bubble-volume-concentration regions, and vice versa. These findings suggest that the fluid blobs containing few bubbles descend in the bubble-rich bulk; it is likely that a water film (heavy fluid) falls in air circumstances (light fluid).

**Figure 5:**
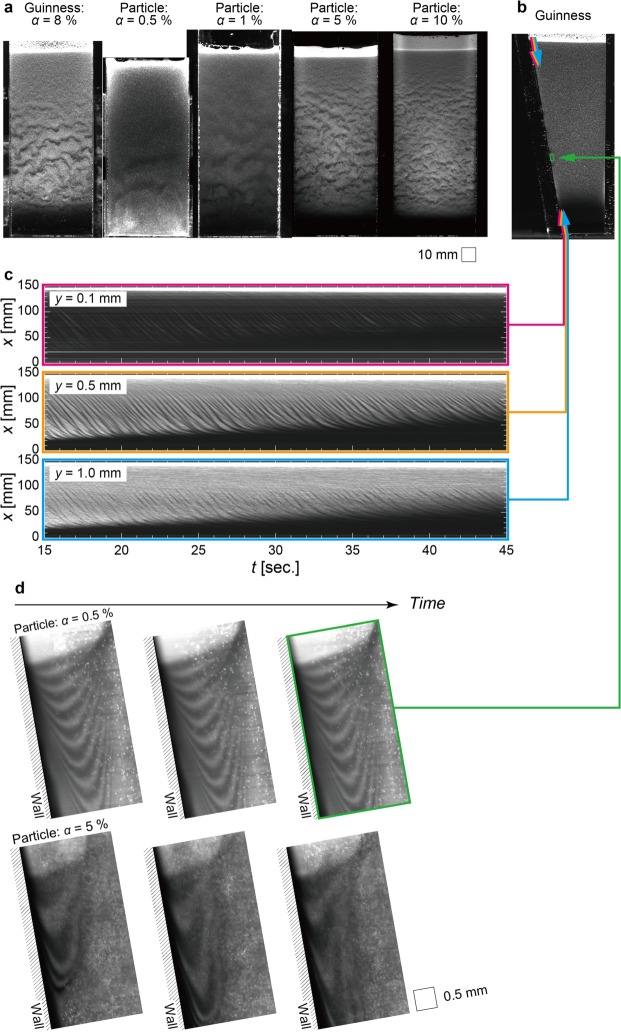
Textures in Guinness and a particle suspension. (**a**) Snapshots of bubble-concentration waves and particle-concentration waves in the trapezium container for various bulk particle concentrations *α*. (**b**) Side view of trapezium container exhibiting uniform bubble distribution, except in the inclined wall vicinity. The corresponding movie is included as Supplementary Movie S3. (**c**) Temporally expanded image of bubble-concentration distribution for various distances from the inclined wall *y*. (**d**) Temporal variations of timeline image visualisation of the liquid phase at *α* = 0.5% (upper) and *α* = 5% (lower). The lines of molecular tag are formed by an intermittently irradiated laser beam where photo-breaching reactions result in dark (i.e., a non-fluorescent) regions.

Here, we consider a particle suspension comprising tap water and hollow glass spherical particles, rather than Guinness containing bubbles, to determine the effect of the volume-concentration of dispersed bodies *α*. The results for the texture-formation in particle suspension with various values of *α* are compared with those in Guinness in Fig. 5a. In the particle suspension, as for the novel findings, we can observe the texture, which is comparable with that in Guinness, at all particle concentrations *α*. The side view of the trapezium container answers the crucial question of where the texture appears in a container. Although bubbles rise in the bulk because of the density difference between the liquid and gas phases, bubbles move downwards in the inclined wall vicinity due to the Boycott effect^8^ (see accompanying Supplementary Movie S3). As also shown in Fig. 5b, for almost the entire region of the trapezium container including the vertical wall vicinity, the texture cannot be observed in the container, except for the downward flow confined to the inclined wall vicinity. We re-plot this finding in temporally expanded images in Fig. 5c for various distances from the inclined wall: *y* = 0.1 mm (top), *y* = 0.5 mm (middle), and *y* = 1.0 mm (bottom). For both *y* = 0.5 mm and *y* = 1.0 mm, we observe a diagonal pattern with a fluctuation in brightness corresponding to the waves travelling downward. For *y* = 0.1 mm, on the other hand, the image includes a darker region corresponding to the so-called clear-fluid (bubble-free) region^7^.

### Movement of liquid phase flow

We attempted to determine the exact liquid-phase velocity profile in this thin clear-fluid film. Timeline-image visualisation applying photo-bleaching molecular tagging method was implemented to characterise the liquid-phase velocity at the wall vicinity, as shown in Fig. 5d. The displacements of timelines, which appear as darker regions (molecular tag), exhibit the following behaviours: unsteady descending flow, a maximum descending velocity at a certain location, zero velocity relative to the boundary, and ascending flow in the interior of the container. Furthermore, the number-density of particles significantly decreases below the location at which the magnitude of the liquid velocity is maximised. This formation of the stratified layer causes a gravity current accompanying the global convection (the Boycott effect), as mentioned above^8^.

**Figure 6:**
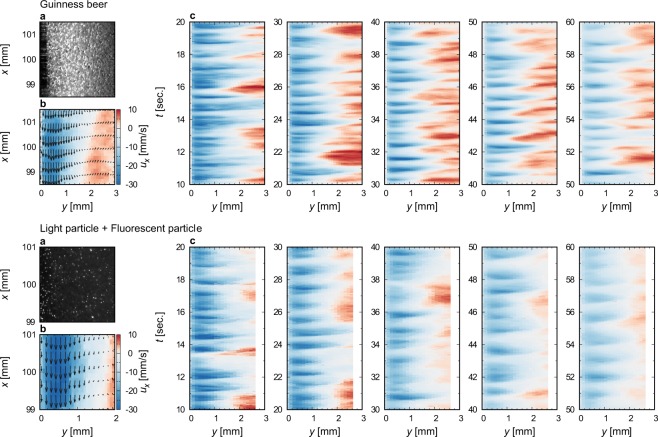
Velocity profiles of liquid phase in Guinness and in a particle suspension. (**a**) A typical snapshot of bubbles or tracer particles. Resin particles with 15 μm in diameter containing laser induced fluorescent (LIF) dye were used as tracer particles. (**b**) An instantaneous velocity vector field. The colour indicates the velocity along the wall. We treated bubbles in Guinness as behaving like tracer particle because of the small relative velocity between a bubble and the surrounding liquid phase as estimated by Eq. C 1. (**c**) Space-time diagrams for descending fluctuations. Velocities along the wall are plotted on the same scale as in (**b**).

The details in the velocity profile of the liquid phase are also obtained via PIV. We apply PIV for Guinness (top) and a particle suspension with *α* ≈ 5% (bottom). From the instantaneous wall-normal variations of the velocity component along the wall, as shown in Fig. 6b, the descending and ascending motion of the liquid phase exhibit identical feature with that obtained from the photo-bleaching visualisation. To address the velocity fluctuations along the inclined wall, we plot a velocity profile at *x* = 100 mm in space-time diagram (Fig. 6c). The magnitude of the descending velocity decreases with increasing time because of the reduced amount of the buoyant bubbles, the velocity fluctuations continue to form. Although we found that there are differences in the magnitude and period in the velocity fluctuations between the Guinness and the particle suspension, we judge that these parameters are consistent with the spatio-temporal characteristics of the waves.

### Critical condition for texture formation

**Figure 7:**
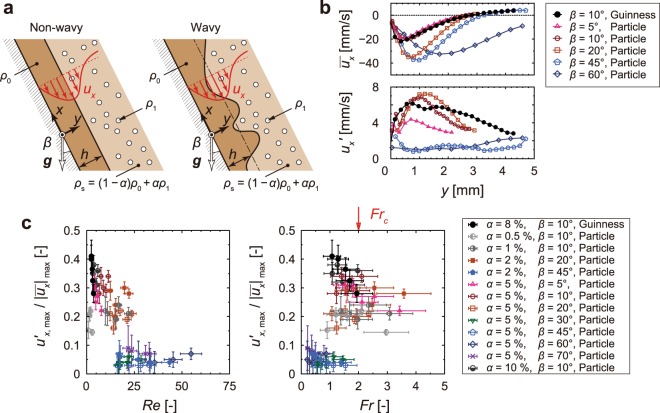
Falling fluid film model for texture-formation. (**a**) Particle separation in the wall vicinity. The fluid can be divided into a clear-fluid region with a density of *ρ*_0_ (above the inclined wall) and a suspension region with a density of *ρ*_s_ = (1 − *α*) *ρ*_0_ + *α ρ*_1_, where *α* and *ρ*_1_ are the volume concentrations of the dispersed phase and the density, respectively. (**b**) Time-averaged velocity along the wall ${\bar{u}}_{x}$ (upper), and root-mean-square value of the fluctuation component ${u}_{x}^{^{\prime} }$ (lower) versus wall-normal distance *y*. (**c**) Scaled velocity fluctuation for various experimental conditions as a function of Reynolds number (left) and Froude number (right). The experimental data are plotted 10 seconds after pouring the fluid and are averaged over 10 seconds at several instants, where error bars indicate resolutions in the measurements.

To obtain an understanding of the texture-formation in the gravity current, we modelled a stratified flow of two liquids: a clear (heavy) liquid film and a bubble-rich bulk (light) liquid, as shown in Fig. 7a. We consider a thin clear-fluid film that flows down on an infinite flat plate of inclination angle *β* with respect to the gravity due to the density difference between the clear and bubble-rich fluid. In a stable flow, the gravity or viscous force tends to keep the interface flat (left panel of Fig. 7a). The bubble-texture forms as an interfacial wave (right panel of Fig. 7a) between the two fluids when the fluid motion becomes unstable. The texture-formation is reflected in the velocity fluctuation. We determined the maximum values of the time-averaged velocity along the wall ${|{\bar{u}}_{x}|}_{{\rm{\max }}}$, the maximum root-mean-square values of the velocity-fluctuation component along the wall ${u}_{x,\,{\rm{\max }}}^{^{\prime} }$, and the loci *h* relative to the wall at which ${|{\bar{u}}_{x}|}_{{\rm{\max }}}$ was achieved via particle image velocimetry (Fig. 7b). Note that it is nontrivial to define the film thickness because of no *sharp* interface between the clear and bubble-rich fluids due to the discrete bubble distribution. In the present study, the film thickness *h* is evaluated from the velocity profile instead of the bubble distribution. Following ref.^19^, in which the particle sedimentation was theoretically studied, the definition of *h* is the distance from the wall, at which the falling liquid velocity takes the maximum and thus the free-slip condition is likely to be satisfied similar to the conventional roll-wave model^18^ with a free-slip interface. It should be noticed that from preliminary measurements of time-averaged profiles of the velocity and the particle concentration *α*, the velocity-based film thickness *h* has been confirmed to be comparable to the distance from the wall, at which *α* takes the half of the particle concentration in the bubble-rich fluid, and thus regarded as a natural choice to characterise the flow. Consequently, from these characteristic quantities, we obtain the Reynolds number and the Froude number expressed respectively as

1 $$Re=\frac{(1-\alpha )\rho \,h\,{|{\bar{u}}_{x}|}_{max}}{(1+\frac{5}{2}\alpha )\mu },$$

2 $$Fr=\frac{2{|{\bar{u}}_{x}|}_{{\rm{\max }}}}{3\sqrt{\frac{{\rho }_{0}-{\rho }_{1}}{{\rho }_{0}}\alpha \,h\,g\,\sin \,\beta }},$$

where *g* is the gravity acceleration. *Re* is the ratio of the inertia force to the viscous force and is an essential indicator for the transition from laminar to turbulent motion of flows due to the shear instability^20,21^. The fluid motion is stable (laminar) at very low *Re*, whereas it becomes turbulent beyond the critical value *Re*_c_, e.g., *Re*_c_ ≈ 1200 for the boundary layer on a flat plate (Blasius profile)^22^. *Fr* is the ratio of the inertia force to the gravity force and is an indicator for the onset of the roll-wave in a liquid-film^18^. The fluid motion of a falling liquid-film in an inclined open channel is described by the magnitude of *Fr*. At very low *Fr*, the fluid motion finds gravity-driven Poiseuille (laminar, non-wavy) flow. At *Fr* ≥ *Fr*_c_, the flow becomes unstable, and thus waves appear at the free surface owing to the instability of the gravity current. Figure 7c shows the scaled velocity fluctuation ${u^{\prime} }_{x,{\rm{\max }}}/{|{\bar{u}}_{x}|}_{{\rm{\max }}}$ as a function of *Re* and *Fr*. Note that we performed experiments systematically parametrizing *α* and *β* on the basis of *α* = 5% and *β* = 10 degrees, but a few semi-systematic parameters were chosen for an extensive investigation. We resolved the velocity profile 10 seconds after pouring to eliminate the flow disturbance induced by pouring a test fluid. An inclined rectangular container was used to minimise the effect of the container shape on the flow. We plot several instances of a value averaged over 10 seconds. Note that both *Re* and *Fr* decrease with increasing time in all observations because of the decrease of the buoyant force (convection velocity) due to the accumulation of the dispersed bodies at the free surface (see Fig. 6c). Figure 7c shows that ${u^{\prime} }_{x,{\rm{\max }}}/{|{\bar{u}}_{x}|}_{{\rm{\max }}}$ gradually decreases with increasing *Re*, and weakly depends on *Fr* up to *Fr* ≈ 1, but for *Fr* > 1, ${u}_{x,\,{\rm{\max }}}^{^{\prime} }/{|{\bar{u}}_{x}|}_{{\rm{\max }}}$ rapidly increases, i.e., the magnitude of the velocity fluctuation increases owing to the texture-formation, implying that the texture-formation is caused by the roll-wave instability rather than the shear instability.

## Discussion and Conclusion

We have found that the velocity fluctuation depends on *Fr*. The velocity fluctuation should be zero for the laminar regime of a falling liquid film comprising two immiscible fluids (e.g., air and water). However, our result reveals non-zero velocity fluctuations at *Fr* < 1 due to the disturbance induced by the rising bubbles or particles with a terminal velocity of *v*_*St*_ ≈ 1.2 mm/s. A striking feature is that the rapid increase in ${u}_{x,\,{\rm{\max }}}^{^{\prime} }/{|{\bar{u}}_{x}|}_{{\rm{\max }}}$ at *Fr* > 1 is in excellent accord with the onset of the primary instability in the roll-wave, namely, *Fr*_c_ = 2^18^, as theoretically estimated. Bubbles or particles rise by their buoyant force, and thus a clear-fluid film is created. The distribution of the dispersed bodies has a concentration gradient (i.e., indistinct concentration interface) owing to the hydrodynamic diffusivity^23^ or the particle-size distribution^24^ in suspensions. Since the presence of bubbles within the clear-fluid film reduces the density difference between the clear-fluid film and bubble-rich bulk, our oversimplified interpretation of the fluid separation may result in underestimation of *Fr*. The effect of fluctuation in the motion of bubble-rich bulk, hysteresis effect^25^ with decreasing *Fr*, or the finite-size effect^26,27^ of the system on a decaying disturbance convected by the large-scale circulating flow should also be reflected in the texture-formation in the subcritical-state regime (*Fr* < *Fr*_c_). The criterion for the texture-formation is successfully determined by our interpretation of flow, despite the imperfections in the experiment.

Finally, we also performed experiments on light particles with a different mean diameter of 34 μm or 75 μm. Even for the variation in the Stokes rise velocity of the dispersed bodies, the texture-formation and descending motion of bubbles were visually observed well. However, in a glass of Budweiser beer, carbonated water, or champagne, which contains sub-millimetre-sized bubbles (300−500 μm)^1–3^, we cannot observe the texture-formation and the descending motion of bubbles, i.e., characteristic flows in Guinness. Large bubbles rise rapidly, and thus they cannot be trapped into a downward flow by the liquid-phase convection, suggesting that the criterion for the texture-formation also depends on the bubble diameter. To comprehend the generation of liquid phase convection depending on the bubble size is a challenge for future studies and is key to control bubbly flows in a brewing apparatus as well as in a pint glass.

## Supplementary information

Supplementary Information

Supplementary Video S1

Supplementary Video S2

Supplementary Video S3
