# Supplementary material for: Bubble cascade in Guinness beer is caused by gravity current instability
Source: Sci Rep. 2019 Apr 5;9:5718. doi: 10.1038/s41598-019-42094-0 (PMC6450914; doi:10.1038/s41598-019-42094-0)
Supplement: Supplementary file 1 — Supplementary Information [file 41598_2019_42094_MOESM1_ESM.pdf]

Bubble cascade in Guinness beer is caused by gravity current instability

Tomoaki WATAMURA[1 a], Fumiya IWATSUBO[1], Kazuyasu SUGIYAMA[1], Kenichiro YAMAMOTO[2], Yuko YOTSUMOTO[2], Takashi SHIONO[2]

[1] *Graduate School of Engineering Science, Osaka University, 1-3, Mchikaneyama, Toyonaka, Osaka 560-8531, Japan*

[2] *Research Laboratory for Beverage Technologies, Research & Development Division, Kirin Co. Ltd., 1-17-1, Namamugi, Tsurumi-ku, Yokohama, Kanagawa 230-6826, Japan*

[a] tomoaki.watamura@me.es.osaka-u.ac.jp

Appendix A:
Measurement of the physical properties of Guinness beer

To measure the physical properties of the liquid medium, we prepared degassed Guinness. We sampled 80 g of Guinness into a 100-ml medium bottle. The 8 samples (2 batches from 4 different production lots) were degassed by means of a centrifugal evaporator (Genevac Ltd.), setting the evaporator to 500 G and 100 mbar for 20 minutes. At different temperatures, we measured the kinematic viscosity ν (Fig. S1a) and density ρ (Fig. S1b), 8 times for each sample. The viscosity was measured in the range above 10 °C; therefore, we extrapolated ν = 2.03 mm^2/s using a linear approximation and ν = 2.23 mm^2/s at 5 °C. Then, the viscosity of Guinness was obtained as $\mu = \nu / \rho$. To measure the bubble volume-concentration, we poured Guinness into a 210-ml measuring cup that overflowed within 10 seconds after opening a can. The foam above the cup was removed with a spatula, and drops attached at the outer wall of the cup were wiped immediately. The measuring cup was placed on a digital weight scale, and then the weight of bubbly Guinness was measured. Finally, the bubble volume-concentration (see Fig. S1c) was estimated from the weight ratio of bubbly medium to the degassed Guinness liquid, subtracting the weight of the measuring cup.

Appendix B:
Bubble and particle size distribution

We measured bubble diameter of Guinness beer by means of image analysis. The bubble images were captured by a high-speed video-camera with a microscope lens, in three different experimental runs. The particle images, on the other hand, were taken by a still-camera with a microscope lens. At least 3000 samples were measured in each case. The bubble and particle size distribution are shown in Fig. S2 for various experiments with reported values [1]. The log-normal distribution was found to give a good fit for the bubble and particle size distribution, even though a physical justification remains insufficient. The fit provides a good comparison of the distribution profile. We also found that the bubble size distribution does not show any remarkable change with increasing time. Therefore, we modelled hollow solid particles as bubbles in a pseudo-Guinness fluid.

Appendix C:
Estimation of bubble rise velocity

To compare the convection velocity and the relative velocity of bubbles to liquid phase, we estimated the Stokes rising velocity of a dispersed body as

$$v_{St} = \frac{(\rho_0 - \rho_1) D_b^{\,2} g}{18\mu}, \tag{C 1}$$

where ρ_0, ρ_1, D_b, g, and μ are the density of the liquid phase, density of the dispersed phase, mean diameter of the dispersed body, the gravity acceleration, and viscosity of liquid phase, respectively. Note that we treat a bubble in Guinness as behaving like a rigid sphere, because Guinness comprises water, ethanol, sugar, soluble solids, and other beer components. The physical properties and the terminal rise velocity of the bubbles in Guinness are compared with those previously reported as summarised in Table S1.

References

1. Robinson, M., Fowler, A. C., Alexander, A. J., O'Brien, S. B. G., "Waves in Guinness", *Phys. Fluids,* **20**, 067101 (2008).
2. Benilov, E. S., Cummins, C. P., Lee, W. T., "Why do bubbles in Guinness sink?", *Am. J. Phys.,* **81** (2), 88-91 (2013).

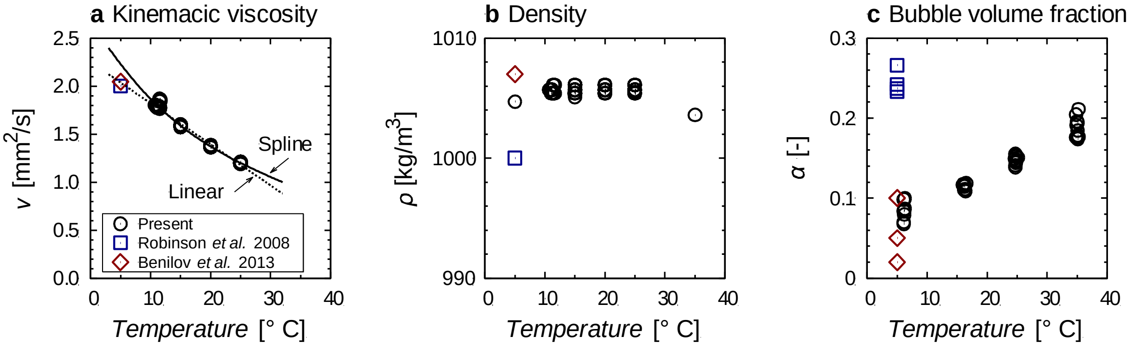

Figure S1 Physical properties of Guinness at various temperatures. **a**, The kinematic viscosity was measured by means of a rolling ball type viscometer (Anton-paar GmbH, Lovis 2000M/ME). Note that we calibrated the viscometer by using degassed water before measurements. **b**, The density was measured by means of an oscillating U-tube-type density meter (Anton-paar GmbH, DMA 35N). **c**, The bubble volume-fraction was measured from the specific gravity of bubbly Guinness.

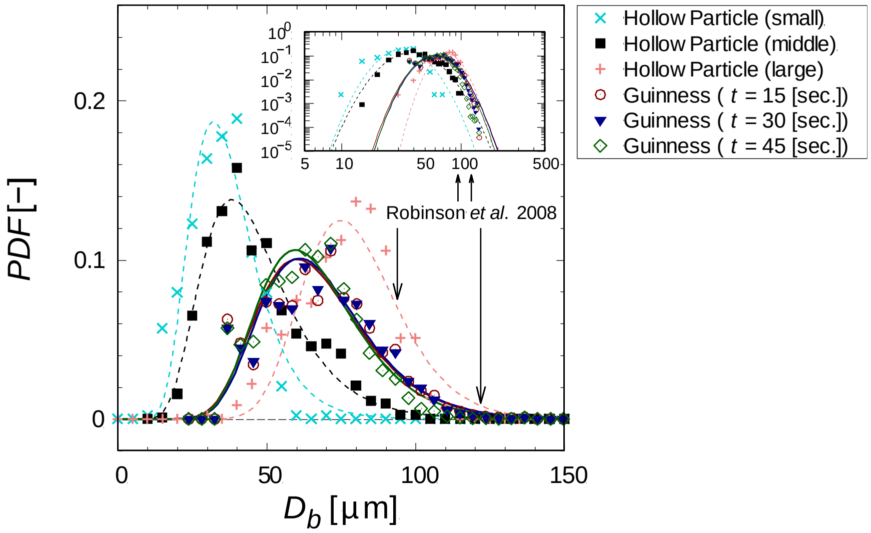

Figure S2 Probability density function (*PDF*) of bubbles and particle diameters. The bubble diameters were measured at three different times after pouring Guinness into a trapezium container, which show relatively small differences. The arrows indicate the mean diameters of bubbles ≈ 94 μm and 122 μm, as reported in the literature [1]. Note that careful attention was paid to removing damaged hollow particles by means of elutriation. We then sifted them to classify the mean and the deviation of hollow particles using sifter baskets. The inset shows a log-log plot of the *PDF* The size distributions are well approximated by the log-normal distributions plotted as lines.

Table S1 Fluid properties of Guinness beer

	Temperature [°C]	Density ρ [kg/m^3]	Viscosity μ [Pa·s]	Volume concentration of the dispersed body α [%]	Mean diameter of the dispersed body D_b [µm]	Stokes' rising velocity v_{St} [mm/s]
Guinness (Robinson *et al.* [1])	5	1007	2×10^{-3}	23.3 – 26.6	94, 122	4.06 – 2.40
Guinness (Benilov *et al.* [2])	6	1007	2.06×10^{-3}	2, 5, 10	122 (referring Ref [1]), 90	3.94 – 2.14
Guinness (present work)	5	1006	2.03×10^{-3} – 2.21×10^{-3}	8	54 – 68	0.716 – 1.15
Particle suspension	25	997	0.89×10^{-3}	0.5 – 10	47	1.20
